# The Potential Role of Concentrated Animal Feeding Operations in Infectious Disease Epidemics and Antibiotic Resistance

**DOI:** 10.1289/ehp.8837

**Published:** 2006-11-14

**Authors:** Mary J. Gilchrist, Christina Greko, David B. Wallinga, George W. Beran, David G. Riley, Peter S. Thorne

**Affiliations:** 1 University Hygienic Laboratory, Iowa City, Iowa, USA; 2 National Veterinary Institute, Uppsala, Sweden; 3 Institute for Agriculture and Trade Policy, Minneapolis, Minnesota, USA; 4 Iowa State University, Ames, Iowa, USA; 5 College of Public Health, The University of Iowa , Iowa City, Iowa, USA

**Keywords:** antibiotic resistance, influenza, manure lagoon, poultry, swine, zoonotic disease

## Abstract

The industrialization of livestock production and the widespread use of nontherapeutic antimicrobial growth promotants has intensified the risk for the emergence of new, more virulent, or more resistant microorganisms. These have reduced the effectiveness of several classes of antibiotics for treating infections in humans and livestock. Recent outbreaks of virulent strains of influenza have arisen from swine and poultry raised in close proximity. This working group, which was part of the Conference on Environmental Health Impacts of Concentrated Animal Feeding Operations: Anticipating Hazards—Searching for Solutions, considered the state of the science around these issues and concurred with the World Health Organization call for a phasing-out of the use of antimicrobial growth promotants for livestock and fish production. We also agree that all therapeutic antimicrobial agents should be available only by prescription for human and veterinary use. Concern about the risk of an influenza pandemic leads us to recommend that regulations be promulgated to restrict the co-location of swine and poultry concentrated animal feeding operations (CAFOs) on the same site and to set appropriate separation distances.

## Background and Recent Developments

As a general principle, the concentration of humans or animals in proximity enhances potential transmission of microorganisms among members of the group. It also creates greater potential for infecting surrounding life forms, even those of different species. The conditions created also may be a breeding ground for new, more infectious, or more resistant microorganisms.

As the human population increases, and mega cities grow, there is greater risk that infectious diseases will evolve, emerge, or spread readily among the populace. The increasing food needs of the growing human population likely will lead to greater populations of livestock. The concentration of animals may augment the risk of zoonoses, diseases transmissible from animals to humans. All segments of livestock production might potentially contribute to zoonotic disease, including transportation of livestock, manure handling practices, veterinary medicine, meat processing and animal rendering. Ideally, everyone involved in each of these components of the industry should be cognizant of the infectious disease risks to animals and humans alike.

Among the many examples of existing risks, some of the more recent are highly pertinent. Nipah virus infections, which occurred in concentrated swine herds in Malaysia and Singapore, killed swine and swine workers (Chua et al. 1999; Paton et al. 1999). Avian influenza has recently infected and caused deaths among poultry and poultry workers in Asia, South America, North America, and Europe [Centers for Disease Control and Prevention 2005; World Health Organization (WHO) 2004]. Many zoonoses may not be related solely to concentrated animal husbandry, but this workshop was devoted to those at least partially attributable to concentration and practices associated with them. While there are many known potential risks for human infection that may result from high concentrations of animals, this article will focus on two—influenza and antibiotic resistance. In addition, we briefly discuss the means of transmission or propagation of infectious agents, including water, animal feed, and human food.

### Antibiotic resistance

#### State of science

Antibiotic resistance is increasing among most human pathogens. The many bacteria resistant to multiple antibiotics in particular has heightened concern. In some cases there are few or no antibiotics available to treat resistant pathogens [Institute of Medicine (IOM) 1998; Mølbak et al. 1999]. Development of new antibiotic classes has lagged behind pharmaceutical innovation in other areas, and some innovative new approaches to combating infection are still immature and unproven (Infectious Diseases Society of America 2005; IOM 1998). Escalating resistance has raised concern that we are entering the “post antibiotic era,” meaning we may be entering a period where there would be no effective antibiotics available for treating many life-threatening infections in humans. If this proves true, deaths due to infection will once again become a very real threat to substantial numbers of children and young adults as well as the sick and the elderly.

Increased antibiotic resistance can be traced to the use and overuse of antibiotics. Much of that use occurs in human medicine. Health care policy and practice changes designed to minimize this phenomenon are in place in many countries, yet much more can be done. Although antibiotic overuse in animals is problematic, the magnitude of the problem is unknown. There is no national mechanism for collecting data on antibiotic use in many countries and the pharmaceutical industry treats production and sales figures as confidential business information. However, the Union of Concerned Scientists (2001) has estimated that 11.2 million kg of the antibiotics used annually in the United States are administered to livestock as growth promoters. This compares with their estimate of 1.4 million kg for human medical use. Their estimates indicate that 87% of all antibiotic use is for animals, while 13% is for human therapeutic and nontherapeutic use. One researcher suggests lower figures for antibiotic use in growth promotion, stating that no more than 40% of antibiotics in the United States is for animals (Levy 1998). As the IOM recently concluded,

Clearly, a decrease in antimicrobial use in human medicine alone will have little effect on the current situation. Substantial efforts must be made to decrease inappropriate overuse in animals and agriculture as well. [National Academy of Sciences (NAS) 2003]

Therapeutic antibiotic administration at high levels for the duration of an illness is obviously an important aspect of veterinary care. However, most animal antibiotic use is designed to promote growth and improve feed conversion ratio. However, the growth rate gains with antibiotic growth promotants are less significant with currently used breeds of swine and poultry (Wegener 2003). This prolonged use of antibiotics, especially at low levels, presents a risk of not killing the bacteria while promoting their resistance by selecting for resistant populations. The resistance genes can pass readily from one kind of bacteria to another (Levy 1998). Thus, workers in the animal units may become colonized with resistant organisms and can pass them on to co-workers and family members or friends. Consumers of meat may also become colonized through mishandling of raw meat or through insufficient cooking. Ultimately, these genes may pass into pathogens, and diseases that were formerly treatable will be capable of causing severe illness or death (NAS 2003).

Evidence of resistance associated with antimicrobial growth promotants has been emerging over the past three decades. Tetracycline-resistant organisms were found in 1976 in chickens raised on feed supplemented with tetracycline, a human-use antibiotic. In a prospective study of 11 poultry farm members and 24 neighbors, Levy and co-workers (1976a) found that before the use of tetracycline on the farm neither the farmers nor the animals were positive for tetracycline-resistant intestinal flora. Within 5 months of the introduction of tetracycline in the poultry feed, 31.3% of fecal samples from farm members harbored intestinal flora that were resistant to tetracycline even though none had been treated clinically with tetracycline. Tetracycline-resistant bacteria were found in only 6.8% of the samples from neighbors. Vancomycin-resistant enterococci arose in livestock in Europe in the 1970s because of use of Avoparcin as an antibiotic growth promotant. Neither Avoparcin nor vancomycin was approved for use in livestock in the United States, and vancomycin-resistant enterococci did not emerge in U.S. livestock (Levy et al. 1976b). White and co-workers purchased 200 samples of ground meat in the Washington, DC, area and found that 20% contained culturable *Salmonella*. Of these, 84% of the organisms were resistant to at least one antibiotic tested, and 53% were resistant to three or more (White et al. 2001). Tetracycline resistance genes were identified in a swine CAFO and also in the manure lagoon serving that CAFO and in ground-water 250 m downstream of the lagoon (Chee-Sanford et al. 2001). Using a medicated feed containing tylosin (a macrolide antibiotic), Zahn et al. (2001) compared swine CAFOs with CAFOs using a nonmedicated feed and observed a 3-fold higher concentration of tylosin-resistant bacteria in the exhaust air from the CAFOs. Antibiotics have also been measured in the dust from swine CAFOs (Hamscher et al. 2003).

Several recent studies clearly demonstrate the transmission of multidrug-resistant pathogens from swine to humans. A French group studied 44 nasal *Staphylococcus aureus* isolates from healthy pig farmers and 21 healthy controls. Five isolates were found in pig farmers that were methicillin resistant. Other isolates were resistant to penicillin, lincomycin, erythromycin, pristinamycin, kanamycin, pefloxacin (Armand-Lefevre et al. 2005). By comparing these findings with analyses of isolates from swine infections, the authors concluded that transmission of these resistant organisms from swine to pig farmers may be frequent. Voss and co-workers (2005) in the Netherlands studied methicillin-resistant *S. aureus* (MRSA) among 26 Dutch farmers living nearby a sentinel case of MRSA. Their study demonstrated transmission of three strains of MRSA from swine to pig farmers, from pig farmers to their family members, and from a hospitalized patient (the sentinel case) to a nurse. Investigators in the United States collected air samples via liquid impingers in a swine CAFO and analyzed the samples for viable isolates of antibiotic resistant bacteria (Chapin et al. 2005). Enterococci, staphylococci, and streptococci were analyzed for resistance to erythromycin, clindamycin, virginiamycin, tetracycline, and vancomycin. None of the isolates were resistant to vancomycin, which has never been approved for use in livestock in the United States. In contrast, 98% of the isolates displayed resistance to two or more of the other four antibiotics that are commonly used as growth promotants in swine. It is important to note that 37 of 124 isolates were resistant to all four of these antibiotics (Chapin et al. 2005).

Sweden banned the use of antibiotics as feed additives for growth promotion in 1985 (Swedish Veterinary Antimicrobial Resistance Monitoring 2003). At that time Sweden used 20 metric tons of antibiotics for growth promotion, 14 metric tons for group treatment and 17 metric tons for treating individual sick animals. In 2003, with no use allowed for growth promotion, the amount of antibiotics used for group treatment was 2 metric tons (down from 14 metric tons), accompanied by a decrease, rather than an increase, of individual treatment use from 17 to 14 metric tons. This demonstrates that the banning of growth promotants did not lead to increased antibiotic use in other categories. In Denmark, veterinary researchers observed a 74% incidence of vancomycin-resistant *Enteroccocus faecium* in broiler chickens in 1995. Following a 1997 ban, the level of resistance fell to 2% by 2000 [Aarestrup et al. 2001; Danish Integrated Antimicrobial Resistance Monitoring and Research Programme (DANMAP) 2004]. In the European Union, antibiotics also used for human medicine were removed from animal use in 1998, and all use of antibiotics as growth promotants are being phased out by 2006 (Casewell et al. 2003). Currently, Sweden and Denmark use less than 3 g of antimicrobial agents per pig slaughtered, whereas the United States uses 47 g (WHO 2003). The experience from the antibiotic bans for broiler chickens demonstrates that the decrease in production—in terms of decreased feed efficiency—is small and is offset by the savings in the cost of antimicrobial growth promotants (Wegener 2003). According to the WHO the increased cost to producers of producing pigs without antibiotic growth promotants is approximately 1% (WHO 2003) and should be compared with the “likely human health benefits to society of antimicrobial growth promoter termination” (WHO 2003).

Animal crowding, CAFO hygiene, temperature and ventilation control, and stress all have an impact on growth rate and the ability of animals to resist disease. Research on the use of other treatments such as probiotics and vaccines holds promise. Probiotics involve the deliberate use of harmless or even beneficial colonizing organisms in food production. It will be important to provide solutions for the spread of antibiotic resistance via air, water, and direct contact to CAFO workers.

The WHO has called for human and veterinary antimicrobial agents to be sold only under prescription. They have also recommended that all countries establish monitoring programs for tracking use and resistance to antimicrobials. The WHO has also called for a rapid phase-out of the use of antimicrobial growth promotants and the creation of prudent use guidelines for veterinary care (WHO 2003).

These practices are not limited to CAFOs. However, it is widely recognized that antibiotic resistance can be staunched only if every effort is made to limit inappropriate use, both with humans and animals.

#### Risk assessment

Microbial risk assessment is an evolving discipline. Methods have not been developed for estimating risks associated with more than one antibiotic and one bacterium at a time. This approach does not fully address the reality of the CAFO environment, where animals harbor multiple microbial species that are exposed to multiple antibiotics over the course of their lives. Moreover, the existence of genetic multidrug resistance determinants (e.g., plasmids carrying genes coding for resistance to multiple drugs) means that exposure to one antibiotic may lead to increased reservoirs of multiple other antibiotics as well. The fact that resistance determinants may be transferred from benign to pathogenic bacteria means that exposure of one bacterium to antibiotics today hypothetically could result in manifestation of human disease only months to years later. Reservoirs of resistance may develop relatively rapidly and may not be completely reversible. This suggests that reducing antibiotic usage may not lead to equivalent reductions in resistance among all bacteria of concern. Thus, research should be concurrent with new public policies to reduce antibiotic overuse and ensure the protection of public health.

#### Vaccines

Development of vaccines could reduce our reliance on antibiotics. The timing of vaccine administration with respect to maternal antibody levels in infants should be studied. Human vaccine administration is undergoing a revolution in anticipation of mass vaccination strategies that may be required to respond to a bioterrorism event. These strategies may also be applicable to animals in veterinary disease prevention. Several diseases afflicting livestock require further research, including necrotic enteritis in poultry (and the use of ionophores for coccidiostats); pasteurella respiratory disease, and; swine ileitis and swine dysentery, as well as diseases of swine at weaning.

#### Policy initiatives

A number of policy initiatives should be explored to establish consistent and responsible operating practices as well as to promote a shift in current thinking about the value of antibiotic-free meat products. These policies should address all levels of CAFO operation, from the CAFO operators themselves to local, state, and federal governments, veterinarians, agricultural and pharmaceutical industries, and the scientific research community. To ensure sensible use of antibiotics, these issues should also be included in the curricula of pharmacists, doctors, and other medical providers. Furthermore, patients must be suitably informed on the proper use of antibiotics including safe disposal.

Producers and industry leaders can and should be afforded the opportunity to assume a leadership role in reducing antibiotic overuse. This should be encouraged by identifying existing producers—either domestic or international—who are using no or reduced antibiotics and might assume demonstration projects. Along with this, a mentoring system could be created for the purpose of sharing practices that have proven successful in established CAFOs. For example, partners in Sweden and Denmark—countries that have experienced successful transitions to antibiotic-free meat production—might be visited by demonstration team producers, along with veterinarians from the respective countries. Where possible, Danish immigrants or American producers of Danish descent might be paired with Danish producers and veterinarians. These collaborative efforts would require travel funds and the availability of antibiotic-free feed at market prices for the duration of the project. Costs should be tracked and producers reimbursed at the outset so that the interval of adjustment to the new antibiotic-free regimen is not burdensome.

Measures to improve the domestic market for meat raised without routine antibiotics should be sought to promote its vitality as a marketable commodity in the United States. At the same time, new overseas markets should be identified, and these special U.S. products heavily promoted as imports of value and interest to the global economy. In addition, product labeling could be made more comprehensive and explicit so that consumers can identify the product and make selections according to their value system. In fact, such improvements in labeling could be an integral part of an overall quality assurance program that drives the label.

### Infectious diseases

#### Influenza

Zoonoses can be transmitted via water, air, consumption or handling of meat products, or by direct transmission from animals to humans. Recent work by Myers and colleagues demonstrated significantly elevated seroprevalence of antibodies against H1N1 and H1N2 swine influenza virus in occupationally exposed adults compared with controls without swine exposure (Myers et al. 2006). Odds ratios for swine H1N1 infection were 35.3 for farmers, 17.8 for veterinarians, and 6.5 for meat processors. For H1N2 infection odds ratios were 13.8, 9.5, and 2.7, respectively (all significant).

The transmission of influenza is a continuing concern. Whether it comes to humans from avian species or swine, or from avian species via swine, or perhaps from humans to swine, strains of high transmissibility and pathogenicity are likely to evolve and create another pandemic (Nature 2005; Webster and Hulse 2005). Recent outbreaks in Asia have shown that transmission of infectious agents can arise from small farms raising poultry in proximity to domiciles and to other animals. However, because CAFOs tend to concentrate large numbers of animals close together, they facilitate rapid transmission and mixing of viruses. There is a concern that increasing the numbers of swine facilities adjacent to avian facilities could further promote the evolution of the next pandemic. The swine industry has adopted a set of guidelines to minimize these risks, including *a*) entry of wild birds and rodents into CAFOs should be limited; *b*) untreated surface water that may have influenza viruses from aquatic birds should not be used for washing facilities, and *c*) waterfowl use of farm lagoons should be minimized. Such prudent practices will minimize risk. To avoid their becoming a mixing vessel for swine or poultry viruses with human viruses, CAFO workers should be immunized against influenza routinely, preferably with the killed vaccine.

The best means to limit transmission of influenza may already be inferred from available data. However, new questions may arise as practices change. What distances should be established between CAFOs housing swine and those housing poultry? Is there a definable, small farm size with minimal numbers of animals that may be allowed?

Surveillance programs should be instituted that maintain biosecurity in CAFOs while maximizing the ability to identify and respond to animal and zoonotic disease outbreaks quickly and effectively.

#### Waterborne diseases

Concerns persist about surface and groundwater contamination that may have ecosystem and human health impacts. Optimal siting and improved construction practices of CAFOs would reduce the potential for contamination. Escrow accounts or insurance policies that would ensure restoration of a vacated manure lagoon to previous conditions should be imposed on those considering building a CAFO. Solid tanks or reservoirs rather than earthen waste lagoons and municipal-style waste treatment are needed to prevent manure contamination of surface and groundwater with infectious agents or antibiotic resistance genes.

#### Animal feed containing animal byproducts

Animal feed containing animal tissues and by-products is a major concern, as sporeforming bacteria likely will be present even after processing. Included are feathers, offal, carcasses, bone and blood meal, and nervous system and brain tissue. Gram-negative enterobacteria of the genus *Salmonella* will multiply in the food when it is reintroduced at the feeding unit. *Salmonella* can be transmitted to humans through the slaughtering process. Meat packing and CAFO workers are at greater risk of acquiring infection because of their close access to animals and feed. CAFOs are so large and densely populated that when a pathogen is introduced into the system, it is difficult to eliminate. Biosecurity should be rigorous, and extreme quality assurance systems are warranted in these large operations.

#### Meat for human consumption

Pathogens tend to be amplified in animals raised in CAFOs and, thus, are more difficult to eliminate in meat packing processes. Research is needed to develop better ways of controlling pathogen growth in meat. Studies should investigate measures to control *Salmonella* cycling within a CAFO. Improved hygiene and ventilation may be sufficient measures. Better controls on the food processing environment are also indicated. Organisms can amplify very efficiently in a holding pen containing live animals. Multidrug-resistant pathogens are of grave concern and are more likely to arise in animal feeding operations that rely on nontherapeutic antibiotic use instead of enhanced hygiene, air filtration, biosecurity and disease surveillance. Finally, research needs include developing better means to reduce colonization of animals and meat with *Campylobacter*, *Salmonella*, *Escherichia coli*, and other organisms.

## Workshop Recommendations

### Priority research needs

- Discontinue nontherapeutic use in the United States: The practice of feeding antibiotics to animals as growth enhancers should be phased out in the United States as it has in the European Union and as called for by the WHO, the IOM, and many scientific and public health organizations. Research studies should monitor the discontinuation to ensure that the ban on antibiotic use for growth promotion is not supplanted by increased therapeutic use.
- Surveillance programs: Coordinated nationwide surveillance programs (Aarestrup 2004) should be instituted to fully assess the contribution of antibiotic use in livestock production to the creation of ecological reservoirs of resistance, or the transmission of that resistance to humans.
- Strain identification: Fingerprinting of isolates of antibiotic-resistant bacteria and the resistance elements should be conducted to establish relationships among members of the same species. Results should be used to identify unknown sources of resistance and to track changes in resistance profiles in response to diminished antibiotic use.
- Influenza risk: Countries and states should establish minimum separation distances for swine and poultry facilities to reduce the risk of influenza outbreaks.
- Manure storage and waste processing: Livestock production facilities should incorporate solid tanks for manure storage and municipal style waste treatment to limit microbial and nutrient contamination of surface and groundwater.
